# Outcomes of Selective Versus Routine Gastric Tube Decompression After Gastrectomy for Gastric Cancer with Pyloric Obstruction: A Retrospective Cohort Study

**DOI:** 10.3390/jcm15010276

**Published:** 2025-12-30

**Authors:** Yonghu Xu, Yushi Liu, Pengfei Kong, Yantian Fang, Dazhi Xu

**Affiliations:** 1 Department of Gastric Surgery, Fudan University Shanghai Cancer Center, Shanghai 200032, China; yhxu92222@163.com (Y.X.); 17301050159@fudan.edu.cn (Y.L.); kongpf@fudan.edu.cn (P.K.); 2 Department of Oncology, Shanghai Medical College, Fudan University, Shanghai 200032, China

**Keywords:** gastric cancer, pyloric obstruction, gastrectomy, gastric tube, enhanced recovery after surgery, healthcare costs, nutritional status

## Abstract

**Background/Objectives**: The utility of routine gastric tube (GT) placement following gastrectomy in gastric cancer (GC) patients with pyloric obstruction remains controversial. This practice conflicts with Enhanced Recovery After Surgery (ERAS) principles, and its value in this high-risk subgroup is unclear. This study aimed to compare the clinical and economic outcomes of routine versus selective gastric tube use in these patients, and to identify predictors for prolonged gastric tube retention. **Methods**: A single-center retrospective cohort study was conducted on 133 GC patients with pyloric obstruction who underwent gastrectomy. Patients were stratified into GT (*n* = 63) and non-GT (*n* = 70) groups. Primary outcomes included 30-day complications, 90-day mortality, hospitalization duration, and costs. Univariate and multivariable Cox regression analyses were used to identify predictors of prolonged GT retention. **Results**: Routine GT use provided no clinical benefit, with similar 30-day complication (22.2% vs. 22.9%, *p* = 0.945) and 90-day mortality (1.6% vs. 0%, *p* = 0.290) rates. However, it was associated with a significantly prolonged postoperative hospital stay (8.8 ± 2.5 vs. 8.0 ± 4.2 days, *p* = 0.034) and a mean cost increase of ¥5900 per patient (*p* = 0.006). A dose–response relationship was evident: each additional day of GT retention correlated with 0.57 extra hospital days (r = 0.567, *p* < 0.001) and ¥3600 in added costs (r = 0.360, *p* = 0.004). Multivariable analysis identified longer preoperative fasting time (Adjusted HR = 1.27 per hour, 95% CI: 1.10–1.45, *p* = 0.001) and GLIM-defined malnutrition (Adjusted HR = 2.04, 95% CI: 1.02–4.17, *p* = 0.045) as independent predictors for prolonged GT retention. **Conclusions**: Routine GT placement after gastrectomy in obstructed GC patients increases healthcare costs and prolongs hospitalization without improving clinical outcomes. Preoperative fasting duration and nutritional status are key predictors for prolonged GT need. A selective GT strategy, guided by these parameters, is recommended to optimize recovery and resource utilization, aligning with ERAS principles.

## 1. Introduction

Gastric cancer (GC) remains a formidable global health challenge, with advanced stages often presenting with complications such as pyloric obstruction, which affects about 30% of patients [1,2,3,4]. This mechanical obstruction not only exacerbates malnutrition and delays definitive surgical intervention but also significantly complicates postoperative recovery, leading to ongoing debate regarding optimal perioperative management strategies [5,6,7].

Gastrectomy serves as the cornerstone treatment for alleviating obstruction and restoring gastrointestinal continuity in these patients [8,9]. For decades, the routine placement of a postoperative gastric tube (GT) has been standard practice, intended to provide decompression and enable enteral access. However, this routine practice increasingly conflicts with the principles of Enhanced Recovery After Surgery (ERAS), which aim to minimize surgical stress and accelerate recovery [10,11]. A growing body of evidence in general gastric surgery populations suggests that GT use may paradoxically prolong postoperative ileus and increase the risk of pulmonary complications [11,12,13]. Consequently, contemporary guidelines, including the Japanese Gastric Cancer Treatment Guidelines 2021, now explicitly recommend against routine nasogastric decompression [14].

Despite this shift, a critical evidence gap persists concerning high-risk subgroups, particularly patients with preoperative gastric outlet obstruction. This cohort is frequently excluded from ERAS studies and protocols [14,15] due to their distinct pathophysiology: prolonged obstruction leads to severe malnutrition, gastric wall edema, and profoundly compromised motility, raising legitimate concerns about anastomotic integrity and delayed return of upper gastrointestinal function postoperatively [16,17]. These concerns have perpetuated a specific clinical controversy in this subgroup: while ERAS principles advocate for minimizing tubes, many surgeons remain hesitant to forgo GT placement, fearing an increased risk of complications like anastomotic leak or gastroparesis in these “high-risk” patients. While some guidelines advocate for selective GT application [18], objective criteria to inform this decision in obstructed patients are lacking. Thus, a significant disconnect exists between guideline recommendations for general populations and the pragmatic management of obstructed patients, with no consensus or evidence-based criteria to guide practice.

To address this critical evidence gap, this study was designed to provide the first comprehensive evaluation focused solely on GC patients with pyloric obstruction. We aimed not only to compare the clinical and economic outcomes of routine versus selective GT use in this specific population but also to identify objective preoperative predictors of prolonged GT need. Our study uniquely fills the existing gap by: (1) determining whether the benefits of GT avoidance extend to this high-risk cohort; (2) quantifying the previously unreported economic burden of routine GT use in this setting; and (3) deriving a practical, predictor-based algorithm to guide selective GT placement. Ultimately, by integrating these findings, we propose an evidence-based framework to align the management of these complex patients with ERAS principles, resolve the prevailing controversy, and optimize resource allocation.

## 2. Materials and Methods

### 2.1. Study Design and Ethical Approval

This single-center, retrospective cohort study was conducted at the Department of Gastric Surgery, Fudan University Shanghai Cancer Center (FUSCC). Consecutive patients who underwent gastrectomy between January 2018 and December 2019 were screened for eligibility. The study protocol was reviewed and approved by the Institutional Review Board of FUSCC (Approval No. FUSCC-D-2021-164), and the requirement for informed consent was waived due to the retrospective nature of the analysis. This study was conducted in accordance with the Declaration of Helsinki. The reporting of this study adheres to the STROBE (Strengthening the Reporting of Observational Studies in Epidemiology) guidelines.

### 2.2. Patient Selection

The study enrolled adult patients (≥18 years) with histologically confirmed gastric adenocarcinoma who underwent gastrectomy (curative or palliative) for clinically defined pyloric obstruction. Pyloric obstruction was defined by the presence of at least one of the following: (1) persistent vomiting, (2) radiologic evidence of significant food retention in the stomach, or (3) endoscopic confirmation of stenosis. Key exclusion criteria included: (1) metastatic disease in which the primary treatment strategy for the metastasis was non-surgical (e.g., systemic chemotherapy or targeted therapy) and surgery was not indicated for symptomatic relief of gastric outlet obstruction, (2) incomplete medical records, and (3) postoperative death within 48 h. Eight patients with Stage IV disease were included because they presented with symptomatic gastric outlet obstruction requiring palliative gastrectomy for symptom relief, which was consistent with the study’s focus on surgical management of obstruction.

### 2.3. Data Collection and Variables

Data were retrospectively extracted from electronic medical records using a standardized case report form on the REDCap (Research Electronic Data Capture) platform (Version 10.0.17, Vanderbilt University, Nashville, TN, USA) to ensure data integrity and anonymity. Three independent researchers, blinded to the study outcomes, performed the data collection. Collected variables included:

1. Preoperative variables: Demographic data (age, gender), body mass index (BMI), nutritional status assessed by the Nutritional Risk Screening 2002 (NRS-2002; high risk defined as score ≥ 3) [17], and the Global Leadership Initiative on Malnutrition (GLIM) criteria [18], preoperative fasting duration (calculated from the last oral intake to anesthesia induction), American Society of Anesthesiologists (ASA) physical status classification, and comorbidities. Preoperative dietary status was categorized based on the patient’s ability to tolerate oral intake in the week prior to surgery due to obstruction, as documented in physician and nursing notes: (1) Normal or soft diet: Ability to tolerate solid or soft foods. (2) Liquid diet only: ability to tolerate only liquids (e.g., soup, milk, nutritional supplements). (3) Nothing by mouth (NPO) or intravenous fluids only: inability to tolerate any oral intake, requiring intravenous hydration.
2. Intraoperative variables: Surgical approach (open vs. laparoscopic), extent of lymphadenectomy (D1 vs. D2), estimated blood loss (mL), and reconstruction method (Billroth I, Billroth II, or Roux-en-Y).
3. Postoperative outcomes: The primary outcomes were the duration of GT retention (days; removal criteria: drainage < 400 mL/24 h), total hospitalization costs (adjusted to 2023 Chinese Yuan using a national healthcare-specific inflation index), 30-day postoperative complications, and 90-day all-cause mortality. Laboratory parameters, including nutritional markers (hemoglobin, albumin, pre-albumin) and tumor markers (AFP, CEA, CA19-9, CA50, CA125, CA72-4, CA242), were recorded. Tumor stage was classified according to the 8th edition of the AJCC (American Joint Committee on Cancer) staging manual. Histopathological diagnosis was based on postoperative specimen examination according to standard clinical practice at our institution. The predominant reporting terminology in the included records was “poorly/moderately/well-differentiated adenocarcinoma.” In accordance with common clinicopathological practice. Specific subtypes such as signet-ring cell carcinoma were explicitly recorded when identified.

### 2.4. Standardized Perioperative Management

All patients were managed under a unified institutional clinical pathway to minimize variations in care. Preoperative management for obstructed patients included fasting, nasogastric decompression, and intravenous nutritional support as needed. Postoperatively, patient-controlled analgesia pumps were routinely removed on postoperative day (POD) 2. Urinary catheters were removed on POD 3, and abdominal drains were removed between POD 5–6 if the output was less than 50 mL/day. Standard antibiotic prophylaxis (intravenous cefuroxime 1.5 g at induction) was administered and extended if signs of infection emerged (C-reactive protein > 50 mg/L or procalcitonin > 2 ng/mL). Enteral nutrition via the GT or oral intake was initiated with clear liquids upon the return of flatus (typically around POD 3) and advanced to a semi-solid diet prior to discharge. Discharge criteria included the absence of fever, adequate tolerance of oral intake, and satisfactory pain control with oral analgesics.

### 2.5. Surgical Technique

All surgical procedures were performed by attending surgeons specializing in gastric oncology, each with over five years of experience. The choice of procedure—radical (R0 resection with D2 lymphadenectomy) or palliative gastrectomy—was based on preoperative staging and intraoperative findings. The method of gastrointestinal reconstruction (Billroth I, Billroth II, or Roux-en-Y) was performed according to the surgeon’s discretion and standard institutional practices. The primary surgical devices and equipment used across procedures are specified as follows: Laparoscopic procedures were performed using a standard high-definition laparoscopic system (Karl Storz GmbH & Co. KG, Tuttlingen, Germany). Gastric resection and gastrointestinal reconstruction were routinely performed using linear staplers (Ethicon Inc., Somerville, NJ, USA). Vessel sealing and dissection were accomplished using an ultrasonic energy device (Harmonic ACE^®^+7 Shears, Ethicon Inc., Somerville, NJ, USA) or a bipolar electrothermal vessel-sealing system (LigaSure™, Medtronic plc, Dublin, Ireland). The choice among these specific device models followed the standard inventory and surgical preferences at our institution during the study period. Notably, during the study period, no formal institutional guideline or protocol mandated the use or avoidance of a postoperative gastric tube for patients with pyloric obstruction. The decision to place a GT was at the discretion of the attending surgeon, based on individual patient assessment and surgical judgment.

### 2.6. Statistical Analysis

Continuous variables are presented as mean ± standard deviation if normally distributed or as median and interquartile range (IQR) if non-normally distributed. Normality was assessed using the Shapiro–Wilk test. Group comparisons (GT vs. non-GT) for continuous variables were performed using Student’s t-test or the Mann–Whitney U test, as appropriate. Categorical variables are presented as frequencies and percentages and were compared using the Chi-square test or Fisher’s exact test.

A multivariable Cox proportional hazards regression model with forward stepwise selection (entry criteria *p* < 0.10, retention criteria *p* < 0.05) was employed to identify independent preoperative and intraoperative factors associated with the time to gastric tube removal. In this analysis, the primary endpoint was delayed GT removal, which we defined as GT retention for ≥3 days. This dichotomous cutoff was chosen based on the median retention duration in our cohort. Patients whose GT was removed before postoperative day 3 were considered not to have reached the endpoint (i.e., they did not experience delayed removal); their follow-up time was censored at the time of GT removal. Therefore, in this model, a Hazard Ratio (HR) > 1 indicates a factor associated with an increased hazard (risk) of prolonged GT retention (i.e., delayed removal), while an HR < 1 indicates a factor associated with a decreased hazard of prolonged retention (i.e., shorter time to removal). We acknowledge that the choice of a 3-day cutoff is data-derived and may not be universally applicable; its use here is to facilitate a clinically interpretable analysis of factors associated with prolonged decompression need. Results are reported as hazard ratios (HR) with 95% confidence intervals (CI). A two-tailed *p*-value of <0.05 was considered statistically significant for all analyses. All statistical analyses were performed using SPSS software (version 25.0, IBM Corp., Armonk, NY, USA) and R software (version 4.2.1, R Foundation for Statistical Computing, Vienna, Austria).

## 3. Results

### 3.1. Patient Enrollment and Baseline Characteristics

**Figure 1:**
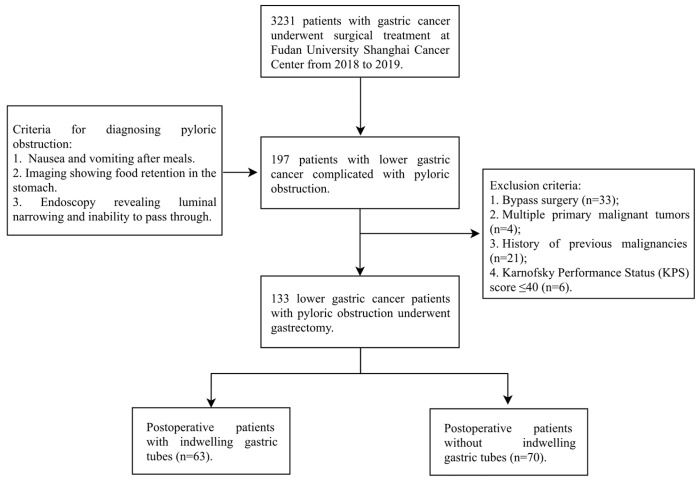
Flowchart of patient selection.

Between January 2018 and December 2019, a total of 133 GC patients with pyloric obstruction who underwent gastrectomy at our institution were included in the final analysis (Figure 1). The cohort consisted predominantly of male patients (67.67%) with a mean age of 61.7 ± 10.9 years. All patients had histologically confirmed gastric adenocarcinoma. Based on the available pathological reports, the cohort comprised: poorly differentiated adenocarcinoma (*n* = 103, 77.4%); moderately differentiated adenocarcinoma (*n* = 2, 1.5%); well-differentiated adenocarcinoma (*n* = 2, 1.5%); signet-ring cell carcinoma (*n* = 26, 19.6%). Regarding lymph node (LN) status, 101 patients (75.9%) had histologically confirmed LN metastasis (pN+). Advanced-stage disease (AJCC Stage II-IV) was present in 85.71% of patients. The vast majority (92.48%) underwent radical resection with D2 lymphadenectomy. The median postoperative hospital stay was 8.64 ± 3.45 days, with average total hospitalization cost of ¥54,100 ± ¥11,700 (Table 1).

Of the 133 GC patients, 63 (47.4%) received a postoperative gastric tube (GT group), while 70 (52.6%) did not (non-GT group). A comparison of baseline characteristics is summarized in Table 2. The two groups were well-matched in terms of demographics, lymph node metastasis, preoperative ASA scores, comorbidities, and Laboratory Findings (Table 3). However, patients in the GT group had a significantly worse preoperative nutritional status. All patients in the GT group (100%) were identified as high nutritional risk (NRS-2002 ≥ 3) and met the criteria for malnutrition according to GLIM (score ≥ 2), compared to only 71.4% (*p* < 0.001) and 15.7% (*p* < 0.001) in the non-GT group, respectively. Intraoperative estimated blood loss was also significantly higher in the GT group (126.6 ± 99.5 mL vs. 93.3 ± 45.5 mL, *p* = 0.019). No other significant differences in surgical or tumor characteristics were observed (Table 4).

### 3.2. Postoperative Complications and Mortality

Routine GT use provided no clinical benefit in terms of postoperative recovery (Table 5). The overall 30-day complication rate was nearly identical between the GT and non-GT groups (22.2% vs. 22.9%, *p* = 0.945). The spectrum of complications, including surgical site infections (4.8% vs. 5.7%), anastomotic leaks (0% vs. 1.4%), and pulmonary complications (3.2% vs. 4.3%), was also comparable. One patient (1.6%) in the GT group died within 90 days due to postoperative sepsis, whereas no mortality was recorded in the non-GT group (*p* = 0.290).

### 3.3. Impact of Gastric Tube Retention Duration on Resource Utilization

**Figure 2:**
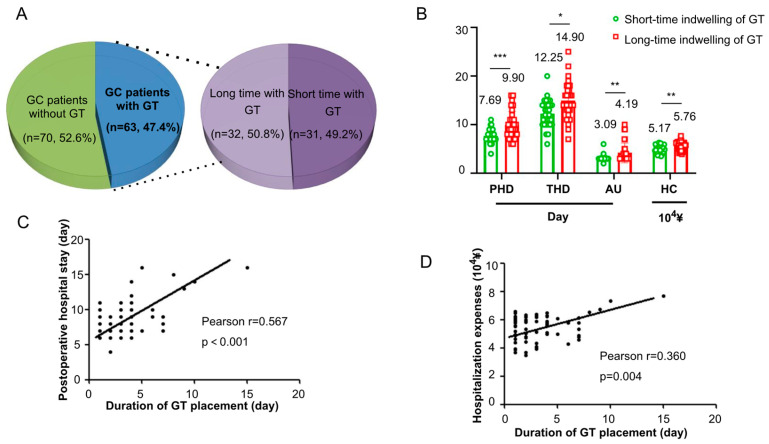
Impact of gastric tube (GT) retention duration on postoperative outcomes. (**A**) Frequency distribution of GT retention duration. The dashed line indicates the median (2 days), used to stratify patients into short-term (<3 days, *n* = 31) and long-term (≥3 days, *n* = 32) retention groups. (**B**) Comparison of postoperative hospitalization duration (PHD), total hospitalization duration (THD), duration of antibiotic use (AU), and total hospitalization costs (HC) between the short-term and long-term GT retention groups. Significance was expressed as: * *p* < 0.05, ** *p* < 0.01, *** *p* < 0.001. (**C**,**D**) Scatter plots showing the correlation between GT retention duration and (**C**) postoperative hospital stay or (**D**) total hospitalization costs.

Among the 63 patients with a GT, the median duration of retention was 2 days (IQR: 1–15 days; mean: 3.35 days). Patients were stratified into a prolonged retention group (≥3 days, *n* = 32) and a short-term retention group (<3 days, *n* = 31) based on the median value (Figure 2A).

Prolonged GT retention was associated with significantly increased resource utilization (Table 6, Figure 2B). Patients in the prolonged retention group had a mean postoperative hospital stay that was 2.2 days longer than those in the short-term group (9.9 ± 2.8 days vs. 7.7 ± 1.4 days, *p* < 0.001). Consequently, the total duration of hospitalization was also significantly extended (14.9 ± 3.8 days vs. 12.3 ± 2.8 days, *p* = 0.024). Furthermore, the prolonged GT group incurred substantially higher hospitalization costs (¥57,600 ± ¥8200 vs. ¥51,700 ± ¥8400, *p* = 0.006) and required a longer duration of antibiotic therapy (4.2 ± 2.0 days vs. 3.1 ± 0.6 days, *p* = 0.006).

**Figure 3:**
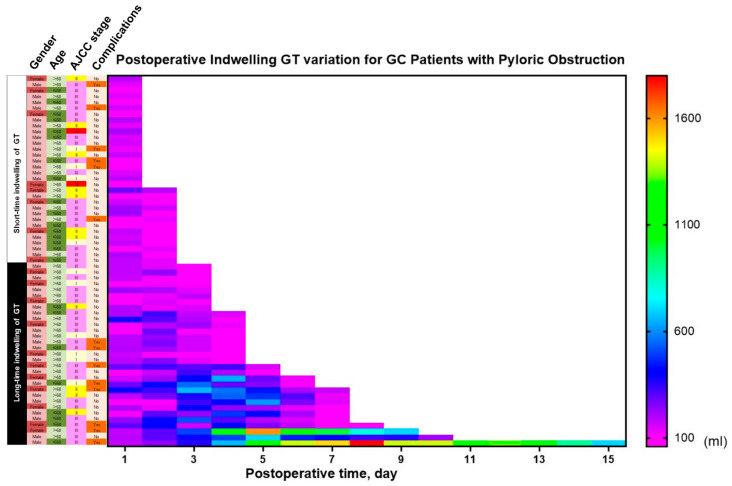
Overview of characteristic differences between patients with long-term (≥3 days) and short-term (<3 days) gastric tube (GT) retention. The heatmap visually summarizes the key preoperative, intraoperative, and postoperative variables that significantly differed between the two groups, highlighting the profile associated with prolonged GT need.

**Figure A1:**
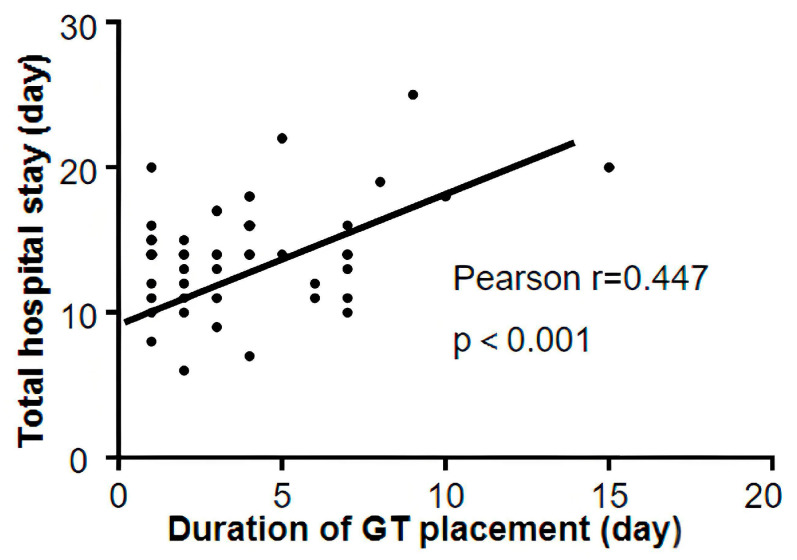
Correlation between gastric tube (GT) retention duration and total hospitalization stay. A scatter plot with a fitted regression line shows a significant positive correlation between the two variables (r = 0.447, *p* < 0.001).

**Figure A2:**
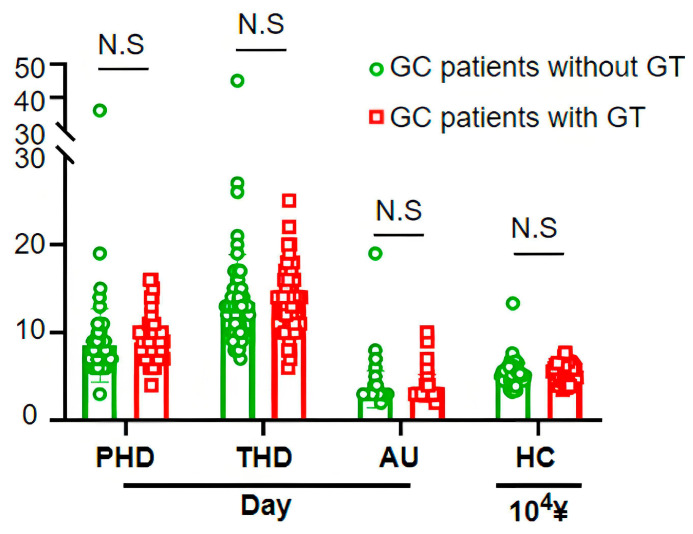
Comparison of postoperative hospitalization duration (PHD), total hospitalization duration (THD), duration of antibiotic use (AU), and total hospitalization costs (HC) between the GT and non-GT groups. Error bars represent standard deviation. N.S, Not significant.

**Figure A3:**
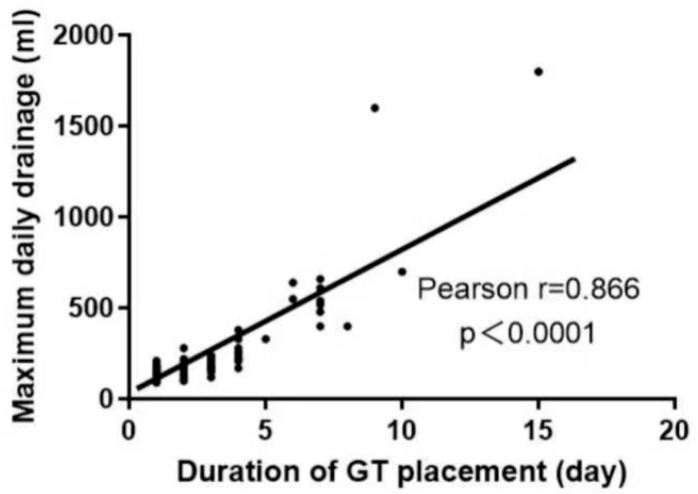
Correlation between gastric tube (GT) retention duration and maximum daily drainage volume. A scatter plot with a fitted regression line demonstrates a strong positive correlation (r = 0.866, *p* < 0.001), indicating that patients with higher daily output required longer GT retention.

A clear dose–response relationship was observed. Each additional day of GT retention was correlated with an extension of the postoperative hospital stay by 0.57 days (r = 0.567, *p* < 0.001; Figure 2C) and an increase in total cost by approximately ¥3600 (r = 0.360, *p* = 0.004; Figure 2D). Additional analyses confirmed strong positive correlations between GT duration and antibiotic use duration, maximum daily drainage volume, and total costs (Appendix A, Figure A1, Figure A2 and Figure A3). An overview of the characteristic differences between the long and short-term GT groups is visually presented in Figure 3.

### 3.4. Economic Burden of Routine Gastric Tube Placement

At the cohort level, the strategy of routine GT placement independently increased healthcare expenditure (Table 7). Even after adjusting for baseline differences, the GT group had a significantly longer postoperative hospitalization (8.8 ± 2.5 days vs. 8.0 ± 4.2 days, *p* = 0.034) and higher total costs per patient (¥54,600 ± ¥9300 vs. ¥48,700 ± ¥13,600, *p* = 0.008). That means patients in the GT group were more likely to incur total costs exceeding ¥59,000.

### 3.5. Predictors of Prolonged Gastric Tube Retention

**Figure 4:**
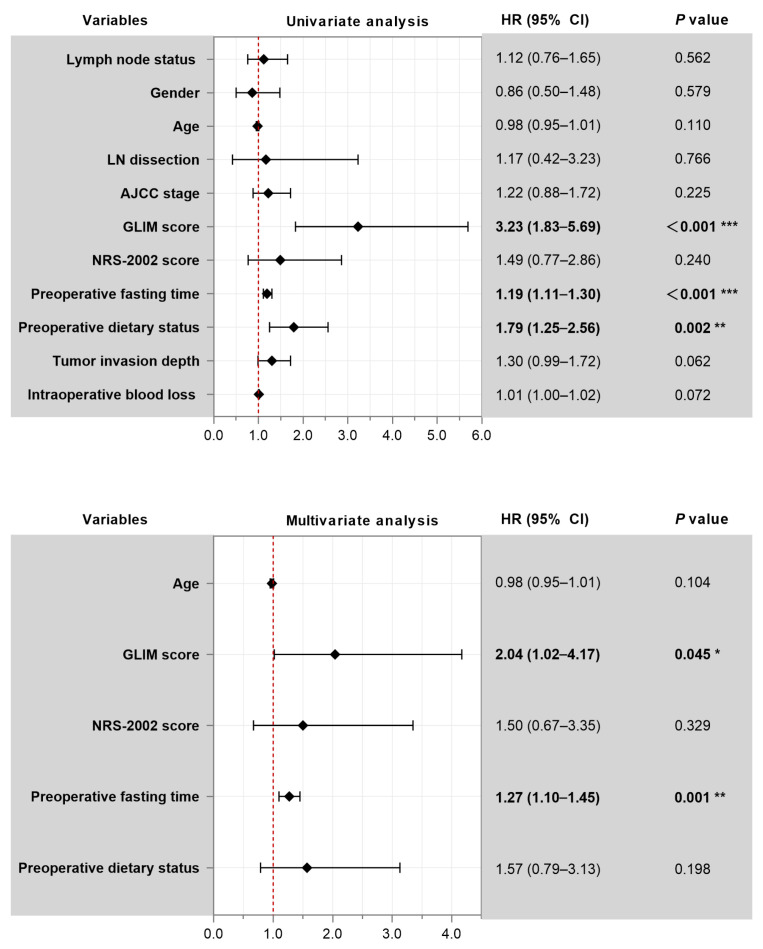
Univariate and Multivariable Analysis of Predictors for Delayed Gastric Tube (GT) Removal. Forest plot displaying the results of univariate (up) and multivariable (down) Cox regression analyses for factors associated with time to GT removal. A Hazard Ratio (HR) > 1 indicates increased risk of delayed removal. CI: confidence intervals; NRS-2002: Nutritional Risk Screening 2002; GLIM: Global Leadership Initiative on Malnutrition. Significance was expressed as: * *p* < 0.05, ** *p* < 0.01, *** *p* < 0.001.

Univariate Cox regression analysis identified several variables associated with an increased risk of prolonged GT retention, including moderate-to-severe malnutrition per GLIM criteria (HR = 3.23, 95% CI: 1.83–5.69, *p* < 0.001), longer preoperative fasting time (HR = 1.19 per hour, 95% CI: 1.11–1.30, *p* < 0.001), and worse preoperative dietary status (HR = 1.79, 95% CI: 1.25–2.56, *p* = 0.002). Tumor invasion depth (HR = 1.30, 95% CI: 0.99–1.72, *p* = 0.062) and increased intraoperative blood loss (HR = 1.01 per mL, 95% CI: 1.00–1.02, *p* = 0.072) also showed a trend toward association. We also evaluated lymph node metastasis as a potential predictor; however, it was not significantly associated with prolonged GT retention in our cohort (HR = 1.12, 95% CI: 0.76–1.65, *p* = 0.562) (Figure 4).

On multivariable analysis, longer preoperative fasting time (Adjusted HR = 1.27 per hour, 95% CI: 1.10–1.45, *p* = 0.001) and the presence of moderate-to-severe malnutrition (GLIM criteria ≥ 2; Adjusted HR = 2.04, 95% CI: 1.04–4.17, *p* = 0.045) were confirmed as independent predictors for an increased risk of prolonged GT retention (Figure 4). This indicates that for each additional hour of preoperative fasting, the risk of prolonged GT retention increased by 27%. Similarly, patients with GLIM-defined malnutrition had a 2.04 times higher risk of prolonged GT retention compared to those without.

## 4. Discussion

This study provides the first comprehensive analysis evaluating the routine use of gastric tubes after gastrectomy specifically in gastric cancer patients with pyloric obstruction, a high-risk population often excluded from ERAS protocols. Our principal findings demonstrate that: (1) routine GT placement failed to reduce postoperative complications or mortality but significantly increased healthcare costs and prolonged hospitalization; (2) a clear dose–response relationship exists between the duration of GT retention and resource utilization; and (3) preoperative fasting duration and nutritional status (assessed by GLIM criteria) are key, easily obtainable predictors for identifying patients at an increased risk of prolonged GT retention. These results challenge the conventional practice of routine GT use in this subset of patients and provide an evidence-based framework for a selective, cost-effective approach.

The absence of clinical benefit associated with routine GT placement aligns with the evolving paradigm in surgical recovery. Our findings—showing nearly identical complication rates (22.2% vs. 22.9%)—are consistent with previous Randomized Controlled Trials (RCTs) and meta-analyses conducted in general gastrectomy populations, which found that avoiding a GT did not increase the risk of anastomotic leakage or other major complications [19,20,21]. This reinforces the concept that routine decompression is unnecessary for most patients, even in the presence of a preoperative obstruction. Crucially, our study extends this evidence base by demonstrating that this principle holds true even in this nutritionally compromised and technically challenging cohort. It is also noteworthy that even short-term decompression strategies fail to confer clinical advantages. A randomized study investigating 1-day nasogastric tube use after distal gastrectomy reported similar complication rates between groups, with no reduction in anastomotic leakage or pulmonary complications, while documenting significant nasopharyngeal discomfort in patients with tubes [22]. This further supports our conclusion that a selective, rather than routine—even if brief—GT approach is warranted.

The most salient finding of our study is the significant economic burden imposed by routine GT use. The additional cost of ¥5900 per patient, coupled with a prolonged hospital stay, represents a substantial and avoidable financial strain on healthcare systems. The strong, quantitative correlations—each additional day of GT retention adding 0.57 hospital days and ¥3600 in costs—provide compelling evidence of a direct causal relationship. This “dose–response” effect underscores that GT retention is not merely a marker of a complicated recovery but is itself a driver of increased resource utilization, likely through mechanisms such as delayed mobilization, increased discomfort, and prolonged ileus [23].

The identification of prolonged preoperative fasting and GLIM-defined malnutrition as independent risk factors for prolonged retention offers critical insights for patient stratification. Each additional hour of preoperative fasting increased the risk of prolonged GT retention by 27%. This likely reflects the intestinal edema and dysmotility induced by prolonged starvation and chronic obstruction, which delay the return of normal upper gastrointestinal (GI) function postoperatively. Similarly, patients with moderate-to-severe malnutrition (GLIM ≥ 2) had a more than two-fold higher risk (HR = 2.04) of requiring prolonged decompression. This highlights the profound impact of a catabolic state on anastomotic healing and gastrointestinal function recovery [24,25,26], necessitating longer periods of decompression and nutritional support. The strong correlation between GT duration and maximum daily drainage volume (r = 0.866, *p* < 0.001) suggests that high-output patients represent a distinct phenotypic subgroup with severely compromised GI function that may genuinely require temporary decompression.

The strong association between GLIM-defined malnutrition and prolonged GT need underscores a critical pathophysiological link. Severe malnutrition, particularly in the context of chronic gastric outlet obstruction, induces a catabolic state characterized by skeletal muscle wasting, including that of the gastrointestinal smooth muscle. This can directly impair gastric and intestinal motility, leading to postoperative ileus and delayed gastric emptying [27]. Furthermore, malnutrition compromises systemic immune function and tissue repair capacity, potentially delaying anastomotic healing and increasing visceral edema, both of which contribute to functional obstruction and the need for prolonged decompression [28,29]. Prolonged preoperative fasting exacerbates this by causing intestinal mucosal atrophy and disrupting the enterohepatic circulation of bile acids, further dampening gastrointestinal hormonal signaling and peristalsis [30]. Therefore, the identified predictors likely serve as proxies for a profound dysfunction of the upper GI tract, explaining the observed dose–response relationship between GT duration and recovery metrics.

**Figure 5:**
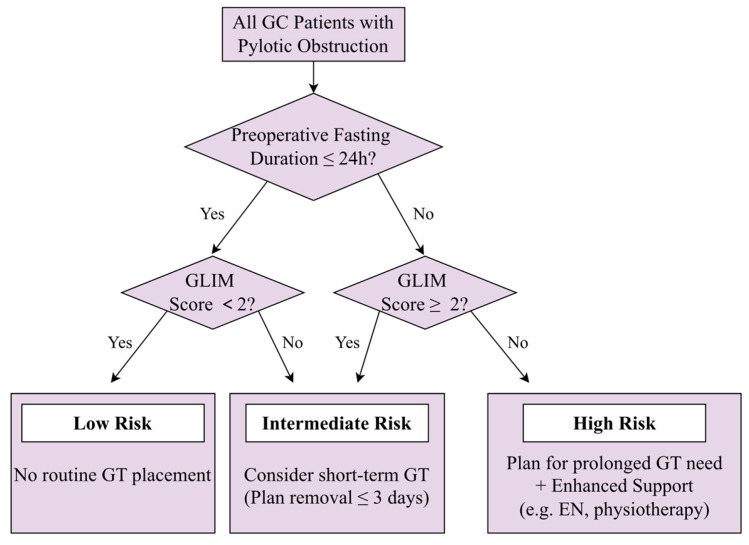
Proposed algorithm for selective gastric tube (GT) use after gastrectomy in gastric cancer patients with pyloric obstruction. Patient stratification is based on the independent predictors of prolonged GT retention identified in this study: preoperative fasting duration and GLIM-defined nutritional status. GT, gastric tube; GLIM, Global Leadership Initiative on Malnutrition; EN, enteral nutrition.

Therefore, a strategy of selective GT placement is warranted. A critical component of this selective strategy is to not only identify whether a GT is needed but also to anticipate for how long it might be required. Our analysis of predictors for prolonged GT retention provides precisely this insight. The fact that GLIM-defined malnutrition and prolonged fasting strongly predict a longer duration of need identifies a patient subgroup in whom GT placement is most likely to be justified and where prolonged retention should be anticipated. Thus, these predictors are not merely prognostic for duration; they serve as key decision-making tools for implementing an overarching selective GT strategy. Based on these insights, we propose a practical, risk-stratified algorithm to guide selective GT use (Figure 5): (1) Avoid routine GT placement in patients with preoperative fasting > 24 h and no malnutrition (GLIM < 2), as they are at very low risk for requiring decompression. (2) Consider short-term GT placement (with a plan for removal within 3 days) for those with either prolonged fasting (>24 h) or moderate-severe malnutrition (GLIM ≥ 2). (3) Anticipate and plan for prolonged GT need in patients with both risk factors (fasting >24 h + GLIM ≥ 2). In this high-risk group, GT placement is justified, and management should include enhanced nutritional support (e.g., early enteral nutrition via the GT) and physical therapy to mitigate the risks of prolonged catheterization. This strategy aligns perfectly with ERAS principles by minimizing unnecessary interventions for low-risk patients while ensuring proactive, personalized management for high-risk individuals.

Our approach to selective GT use aligns with a growing trend in gastrointestinal surgery to tailor decompression practices based on patient-specific risk factors. In general gastrectomy populations, several studies have advocated for omitting routine GT placement, with some proposing selective use in cases of extensive resection, high intraoperative blood loss, or suspected anastomotic tension [19,20]. For instance, Li et al. suggested that GT may be beneficial only in patients with prolonged operative time or significant gastric distension, though their criteria were not explicitly validated in obstructed patients [19]. Similarly, in colorectal surgery, selective nasogastric decompression is often guided by factors such as preoperative bowel obstruction, intraoperative contamination, or surgeon judgment [31,32]. However, these strategies lack standardized, easily assessable preoperative predictors, particularly for gastric cancer patients with outlet obstruction. Our study advances this paradigm by identifying two readily available preoperative variables—fasting duration and GLIM-defined malnutrition—that are specifically relevant to this high-risk subgroup. Unlike previous protocols that rely on intraoperative or subjective clinical assessments, our algorithm provides a structured, preoperative framework that can be integrated into ERAS pathways before surgery, facilitating earlier decision-making and resource planning.

Several limitations inherent in our study design should be considered when interpreting the results. First, as a retrospective single-center analysis, this study is susceptible to selection bias and unmeasured confounding. Although multivariate regression was used to adjust for key baseline differences, the significant disparities in preoperative nutritional status between the groups indicate that the decision to place a GT was non-random and likely influenced by surgeons’ assessment of individual patient risk. Consequently, residual confounding may affect the comparison of outcomes between the GT and non-GT groups. Second, the sample size, while substantial for this specific patient subgroup, may still limit the statistical power for analyzing rare but serious complications like anastomotic leakage. Furthermore, in the Cox regression analysis, the duration of GT retention was dichotomized based on the median value. While this is a common and clinically intuitive approach, the choice of a different cutoff might influence the results. However, the median was chosen for its statistical robustness and representativeness of the cohort’s central tendency. Third, although intraoperative blood loss showed a trend toward association with prolonged GT retention in our univariable analysis, it was not an independent predictor in the multivariate model. This precluded us from incorporating it into our evidence-based algorithm. Future studies with larger sample sizes are needed to validate the potential role of intraoperative factors in predicting the need for gastric decompression. Last, our follow-up was limited to short-term surgical outcomes; long-term assessments of nutritional status and quality of life are needed in future investigations.

Notwithstanding these limitations, our findings provide robust preliminary evidence to inform clinical practice. The proposed risk-stratified algorithm (Figure 5) offers a practical framework for selective GT use. The critical next step is its prospective validation, ideally through a multicenter randomized controlled trial comparing our predictive model-guided selective strategy against both routine placement and surgeon discretion. Such a trial should also incorporate cost-effectiveness analysis and patient-reported outcomes to comprehensively assess its value in integrating obstructed gastric cancer patients into enhanced recovery pathways. Specifically, a pragmatic validation study could be structured as follows: Patients with pyloric obstruction scheduled for gastrectomy would be risk-stratified preoperatively using our algorithm. Those deemed “low-risk” (e.g., fasting <24 h, GLIM < 2) would be randomly assigned to a protocol mandating no GT placement versus current practice. For “high-risk” patients, different strategies of GT management (e.g., early removal protocol vs. standard care) could be compared. Primary endpoints should include comprehensive recovery metrics (time to GI function recovery, complication rates), patient-reported outcomes (discomfort), and economic analyses. Successful validation would require integrating the algorithm into enhanced recovery clinical pathways, with clear decision points and staff education. This stepwise approach from retrospective derivation to prospective validation is crucial for translating our predictive model into a tool that reliably optimizes care and resource use in this challenging patient population.

## 5. Conclusions

In conclusion, this study demonstrates that the routine placement of a gastric tube after gastrectomy in patients with gastric cancer and pyloric obstruction imposes a significant economic burden without conferring any measurable clinical benefit. We have identified preoperative fasting duration and GLIM-defined malnutrition as simple, effective tools for predicting the risk of prolonged GT dependency. Adopting a selective GT strategy guided by these parameters can optimize patient recovery, reduce healthcare costs, and successfully integrate this high-risk population into modern ERAS pathways. Moving away from routine practice towards a precision-based approach is both clinically justified and economically imperative.

## Figures and Tables

**Table 1:** Baseline Characteristics of the Total Study Cohort (*n* = 133).

Characteristics	Value
Age (years, Mean ± SD)	61.70 ± 10.92
Gender (*n*, %)	
Male	90 (67.67%)
Female	43 (32.33%)
Lymph Node Metastasis (*n*, %)	
Present	101 (75.9%)
Absent	32 (24.1%)
Histopathological Type (*n*, %)	
Poorly differentiated adenocarcinoma	103 (77.4%)
Moderately differentiated adenocarcinoma	2 (1.5%)
Well-differentiated adenocarcinoma	2 (1.5%)
Signet-ring cell carcinoma	26 (19.6%)
AJCC 8th Stage (*n*, %)	
I	19 (14.29%)
II	28 (21.05%)
III	78 (58.65%)
IV	8 (6.02%)
Type of Surgery (*n*, %)	
Radical	125 (93.98%)
Non-radical	8 (6.02%)
LN Dissection (*n*, %)	
D1	10 (7.52%)
D2	123 (92.48%)
Postoperative GT Placement (*n*, %)	
Yes	63 (47.37%)
No	70 (52.63%)
Postoperative Hospitalization Duration (days, Mean ± SD)	8.64 ± 3.45
Total Hospitalization Cost (104 ¥, Mean ± SD)	5.41 ± 1.17

SD: Standard Deviation; AJCC: American Joint Committee on Cancer; LN: Lymph Node; GT: Gastric Tube. ¥: Chinese Yuan.

**Table 2:** Patient Baseline Characteristics and Nutritional Status Stratified by Postoperative Gastric Tube Use.

Characteristics	GT Group (*n* = 63)	Non-GT Group (*n* = 70)	*p* Value
Gender (*n*, %)			0.611
Male	44 (69.84%)	46 (65.71%)	
Female	19 (30.16%)	24 (34.29%)	
Age (*n*, %)			0.502
<60 years	36 (57.14%)	44 (62.86%)	
≥60 years	27 (42.86%)	26 (37.14%)	
Lymph Node Metastasis			0.199
Present	51 (81.0%)	50 (71.4%)	
Absent	12 (19.0%)	20 (28.6%)	
ASA score (*n*, %)			0.281
1	34 (53.97%)	41 (58.57%)	
2	19 (30.16%)	24 (34.29%)	
3	10 (15.87%)	5 (7.14%)	
Comorbidities (*n*, %)			0.886
Yes	26 (41.27%)	24 (34.29%)	
No	37 (58.73%)	36 (65.71%)	
NRS-2002 score (*n*,%)			<0.001
<3	0 (0%)	20 (28.57%)	
≥3	63 (100%)	50 (71.43%)	
GLIM score (*n*, %)			
<2	0 (0%)	59 (84.29%)	<0.001
≥2	63 (100%)	11 (15.71%)	

GT: Gastric Tube; ASA: American Society of Anesthesiologists; NRS: Nutritional Risk Screening; GLIM: Global Leadership Initiative on Malnutrition scale.

**Table 3:** Preoperative Laboratory Findings Stratified by Gastric Tube Use.

Characteristics	GT Group (*n* = 63)	Non-GT Group (*n* = 70)	*p* Value
Hemoglobin (g/L)	119.71 ± 27.22	122.81 ± 23.20	0.483
Albumin (g/L)	40.59 ± 4.41	41.15 ± 4.99	0.494
Pre-Albumin (mg/dL)	0.27 ± 0.16	0.24 ± 0.08	0.167
AFP (μg/L)	10.84 ± 48.84	7.62 ± 33.94	0.673
CEA (μg/L)	3.32 ± 2.85	9.78 ± 28.39	0.067
CA199 (μ/mL)	52.42 ± 137.09	72.32 ± 196.95	0.507
CA50 (μ/mL)	11.75 ± 19.63	31.55 ± 87.22	0.073
CA125 (μ/mL)	14.91 ± 12.01	15.39 ± 11.21	0.819
CA72-4 (μ/mL)	8.17 ± 12.87	12.19 ± 40.42	0.440
CA242(μ/mL)	19.36 ± 41.73	23.16 ± 49.48	0.640

GT: Gastric Tube.

**Table 4:** Intraoperative Characteristics Stratified by Gastric Tube Use.

Characteristics	GT Group (*n* = 63)	Non-GT Group (*n* = 70)	*p* Value
Estimated Blood Loss (mL, Mean ± SD)	126.56 ± 99.46	93.29 ± 45.45	0.019
Lymphadenectomy Extent (*n*, %)			0.627
D1	4 (6.35%)	6 (8.57%)	
D2	59 (93.65%)	64 (91.43%)	
Resection Status (*n*, %)			0.622
0	59 (93.65%)	64 (91.43%)	
1	0 (0.00%)	1 (1.43%)	
2	4 (6.35%)	5 (7.14%)	
Reconstruction Method (*n*, %)			0.160
Billroth I	7 (11.11%)	3 (4.29%)	
Billroth II	39 (61.90%)	53 (75.71%)	
Roux-en-Y	17 (26.98%)	14 (20.00%)	

GT: Gastric Tube.

**Table 5:** Postoperative Complications and 90-Day Mortality Stratified by Gastric Tube Use.

Parameter	GT Group (*n* = 63)	Non-GT Group (*n* = 70)	*p* Value
Wound infection	0	2	
Intra-abdominal infection	3	2	
Ileus	0	1	
Chyle leakage	1	0	
Anastomotic leakage	0	1	
Gastroplegia	4	2	
Pulmonary complication	2	3	
Urinary tract infection	2	2	
Hepatic complication	2	3	
Total complications (*n*, %)	14 (22.22%)	16 (22.86%)	0.945
Mortality (*n*, %)	1 (1.59%)	0 (0%)	0.290

GT: Gastric Tube.

**Table 6:** Association Between Gastric Tube Retention Duration and Resource Utilization.

Resource Utilization Measure	Prolonged Retention (≥3 Days, *n* = 31)	Short-Term Retention (<3 Days, *n* = 32)	*p* Value
Preoperative Hospital Stay (days)	5.00 ± 2.39	4.56 ± 1.98	0.434
Postoperative Hospital Stay (days)	9.90 ± 2.83	7.69 ± 1.38	<0.001
Total Hospital Stay (days)	14.90 ± 3.79	12.25 ± 2.77	0.024
Duration of antibiotic usage (days)	4.19 ± 2.02	3.09 ± 0.59	0.006
Total Hospitalization Cost (10^4^ ¥)	5.76 ± 0.82	5.17 ± 0.84	0.006

GT: Gastric Tube; ¥, Chinese Yuan. Data presented as Mean ± Standard Deviation.

**Table 7:** Univariable Analysis of Resource Utilization Stratified by Gastric Tube Use.

Resource Utilization Measure	GT Group (*n* = 63)	Non-GT Group (*n* = 70)	*p* Value
Preoperative Hospital Stay (days)	4.78 ± 2.19	4.99 ± 2.78	0.631
Postoperative Hospital Stay (days)	8.78 ± 2.47	8.01 ± 4.16	0.034
Total Hospital Stay (days)	13.56 ± 3.55	13.50 ± 5.38	0.442
Duration of antibiotic usage (days)	2.98 ± 0.14	2.98 ± 0.13	0.914
Total Hospitalization Cost (10^4^ ¥)	5.46 ± 0.93	5.36 ± 1.36	0.008

GT: Gastric Tube; ¥, Chinese Yuan. Data presented as Mean ± Standard Deviation.

## Data Availability

The data presented in this study are available on request from the corresponding author due to privacy. The data are not publicly available due to containing information that could compromise the privacy of research participants.

## Abbreviations

The following abbreviations are used in this manuscript:

GT	Gastric tube
GC	Gastric cancer
ERAS	Enhanced recovery after surgery
GLIM	Global leadership initiative on malnutrition
HR	Hazard ratio
CI	Confidence interval
FUSCC	Fudan University Shanghai Cancer Center
NRS-2002	Nutritional Risk Screening 2002
ASA	American Society of Anesthesiologists
NPO	Nil Per Os (Nothing by Mouth)
AJCC	American Joint Committee on Cancer
POD	Postoperative day
IQR	Interquartile range
GI	Gastrointestinal
EN	Enteral nutrition
RCT	Randomized controlled trial
